# Pulmonary cement embolism is frequently observed but not a contributing factor for death in patients with cemented total hip and knee arthroplasty: a postmortem study

**DOI:** 10.1007/s00264-022-05381-6

**Published:** 2022-03-29

**Authors:** Jacob Ritter, Jan Hubert, Inga Kniep, Frank Timo Beil, Tim Rolvien, Klaus Püschel

**Affiliations:** 1 Department of Legal Medicine, University Medical Center Hamburg-Eppendorf, Butenfeld 34, 22529 Hamburg, Germany; 2 Department of Trauma and Orthopaedic Surgery, Division of Orthopaedics, University Medical Center Hamburg-Eppendorf, Martinistr. 52, 20246 Hamburg, Germany; 3 Department of Osteology and Biomechanics, University Medical Center Hamburg-Eppendorf, Lottestr. 59, 22529 Hamburg, Germany

**Keywords:** Pulmonary cement embolism, Bone cement, Hip arthroplasty, Knee arthroplasty, Cause of death

## Abstract

**Purpose:**

Bone cement is frequently used for implant fixation in orthopaedic surgery. The occurrence of pulmonary cement embolism (PCE) in hip and knee arthroplasty has been described previously, but the exact extent and frequency have not been adequately studied. A postmortem cohort provides a unique opportunity for a more detailed analysis of this phenomenon.

**Methods:**

Through retrospective analysis of whole-body computed tomography (CT) scans and autopsy protocols, we identified 67 cases with previous cemented total hip or knee arthroplasties. A grading system originally developed for PCE after cemented spine procedures was used. Findings were compared with two control groups: 35 individuals with previous cementless total joint arthroplasty as well as 25 individuals without evidence of prostheses.

**Results:**

PCE was detected in 46.3% of the cases: grade 1 (31.3%), grade 2 (10.5%), and grade 3 (4.5%). No statistically significant difference was found between hip and knee arthroplasties in terms of PCE frequency. Importantly, none of the autopsy reports listed PCE as a cause of death or a contributing factor for the patients’ death. In the two control groups, only one case per group was classified as grade 1 PCE, while the remaining cases did not show any evidence of PCE.

**Conclusion:**

The presented data reveal a high frequency of PCE in hip and knee arthroplasties, which is almost identical to previous findings in patients with cement-augmented interventions in the spine. This way, our results underline the relevance of PCE after arthroplasty, suggesting an adaptation of surgical methods to minimize this complication.

**Supplementary Information:**

The online version contains supplementary material available at 10.1007/s00264-022-05381-6.

## Introduction

Bone cement (polymethylmethacrylate, PMMA) is often used in orthopedic and trauma surgery. Even though the fields of application are diverse, it is often related to prosthesis fixation in total joint replacement and vertebral height restoration after fractures (e.g., vertebroplasty). Bone cement is a mixture of a solid and a liquid component, which hardens under the generation of heat (i.e., exothermic reaction). During application, various complications may occur such as local cement leakage, pulmonary cement embolism (PCE), and haemodynamic compromise [1]. While most PCEs are likely to be small and not haemodynamically relevant, there are also rare cases with large, fatal PCEs that can lead to death [2, 3]. In 2018, we reported the incidence of cement leakage and PCE during spine interventions [4]. Probably due to low awareness, there are no studies investigating the frequency of these complications (leakage and PCE) in the context of hip and knee arthroplasty.

We hypothesized that the PCE rate might be similar for endoprosthetic procedures compared with vertebral cement augmentations, albeit with less severe PCE grades. We further hypothesized that low-grade PCE may not be the cause of death in affected patients. With regard to these hypotheses, we retrospectively examined a postmortem cohort of 67 patients with previous endoprosthetic operations of the lower extremity (i.e., total hip or knee arthroplasty). We focused on both a morphological quantification of PCE via CT and the survival time of the affected patients. We also aimed to correlate the results with the autopsy findings and to compare them with the frequency of PCE within the previously published cohort of patients with vertebral cement augmentations.

## Materials and methods

### Study cohort

We performed a retrospective analysis at the local Department of Legal Medicine of all autopsy protocols and whole-body CT scans from the years between 2009 and 2020. Only those cases with described previous arthroplasty, either of the hip and/or knee, and available CT were included in this cohort. Cases with cement-augmented spine procedures were excluded explicitly. We detected 119 cases with potential arthroplasty and analyzed available CT scans with regard to the use of bone cement for prosthesis fixation. This analysis was performed using the OsiriX® DICOM viewer (OsiriX MD® for mac, v.9.5.2.). Those cases in which cementless prosthetic fixation was performed were excluded. Finally, we identified 67 patients with a total of 89 cemented arthroplasties. The cause of death and clinical information about the time interval between arthroplasty and death were collected and analyzed retrospectively. To validate the results, we further compared the findings with 35 randomly selected patients with available CT scan and detected cementless total joint arthroplasties (clTJA) as well as 25 randomly selected patients with no signs of the previous arthroplasty (control) of similar age, sex ratio, and autopsy year. A retrospective analysis was conducted, and data were anonymized before further analysis [5].

### Radiologic assessment

**Fig. 1:**
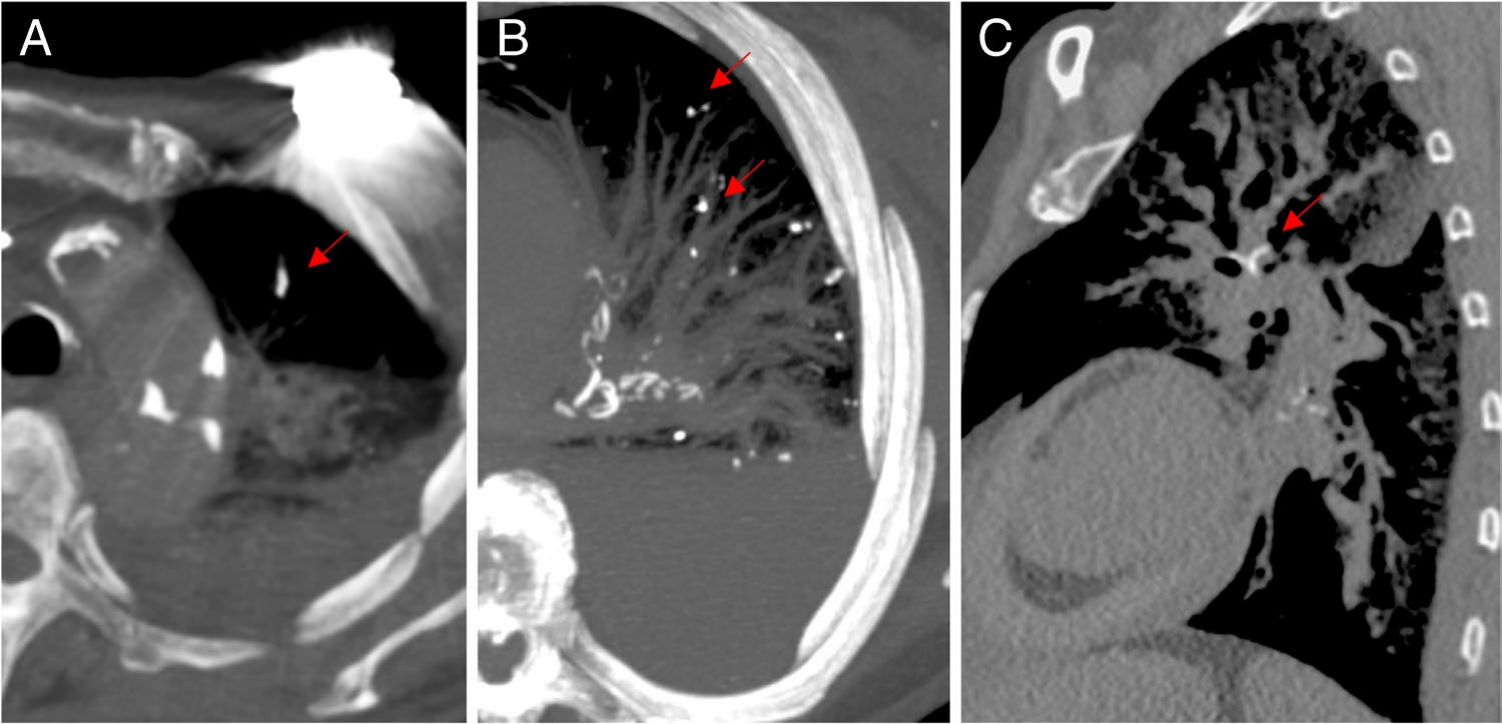
Exemplary chest CT images (bone window) with hyperdense structures classified as grade 3 pulmonary cement (PCE). **A** Axial reformat with signs of a slightly branched PCE. **B** Axial reformat with characters from multiple PCEs. **C** Sagittal reformat with a branched PCE. The red arrows point to the PCE

We suspected that particularly small PCE may occur in hip and knee arthroplasties. Therefore, the CT examinations were performed with a slice thickness of 1.0 mm or lower (Philips Brilliance 16, Philips, Amsterdam, The Netherlands). The evaluation of the images took place in consultation with an expert radiologist. For this purpose, 2D multiplanar reconstruction (coronal, axial, and sagittal view) was chosen and displayed in the lung window (width: 1400 HU; height: 500 HU), abdomen window (width: 350 HU; height: 40 HU), as well as in the bone window (width: 1500 HU; height: 300 HU). PCE was assessed by examining lung slices for hyperdense structures that showed attenuation of > 500 HU. False-positive findings were avoided by excluding hyperdense structures within the distance of 2 cm to the pleural cavity and mediastinum (i.e., coronary arteries and pulmonary hilus). To allow a comparison between the findings in our arthroplasty cohort and our cement-augmented interventions in the spine cohort from 2018 [4], we chose to graduate the new findings in the same manner as grade 0 (no PCE), grade 1 (1–3 PCE), grade 2 (4–6 PCE), and grade 3 (> 6 PCE, or prolonged, branch-shaped cement deposits) (Fig. 1A–C). Furthermore, there are reports in the literature of periprosthetic cement leakage [6], which is why we also analyzed the cohort with regard to the frequency of this complication.

### Statistical analysis

Statistical analyses were performed with GraphPad Prism (Macintosh Version 9.2.0 GraphPad Software, Inc., La Jolla, CA, USA). While we used an unpaired two-tailed *t*-test for comparison of two groups, ordinary one-way ANOVA with Tukey’s multiple comparison test was performed to compare more than two study groups. The level of significance was defined as *p* < 0.05. Exact *p*-values are reported unless *p* < 0.0001. All data are presented as absolute values or as the mean ± standard deviation (± SD).

## Results

**Fig. 2:**
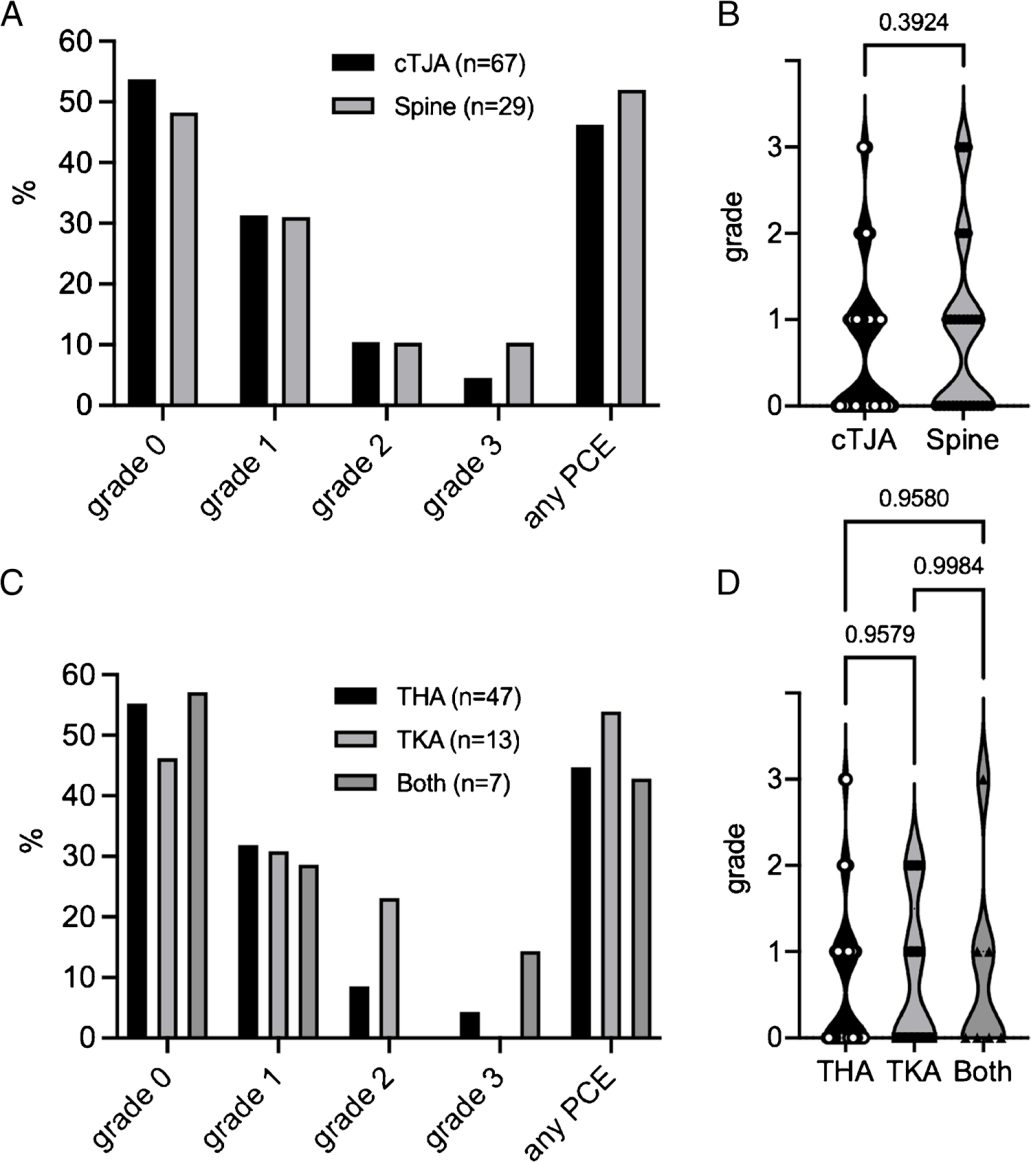
Distribution of PCE according to grades and different surgical procedures. **A** Breakdown of the different PCE grades for cTJA compared to the previously reported cohort with cement augmented spine surgery. **B** Comparison of the mean PCE grade of cTJA with the spine. A *t*-test was used for statistical comparison. **C** Breakdown of PCE grades by THA, TKA, and both. **D** Comparison of the mean PCE grade between THA, TKA, and both. Ordinary one-way ANOVA with Tukey’s multiple comparison test was performed to compare the three groups

**Fig. 3:**
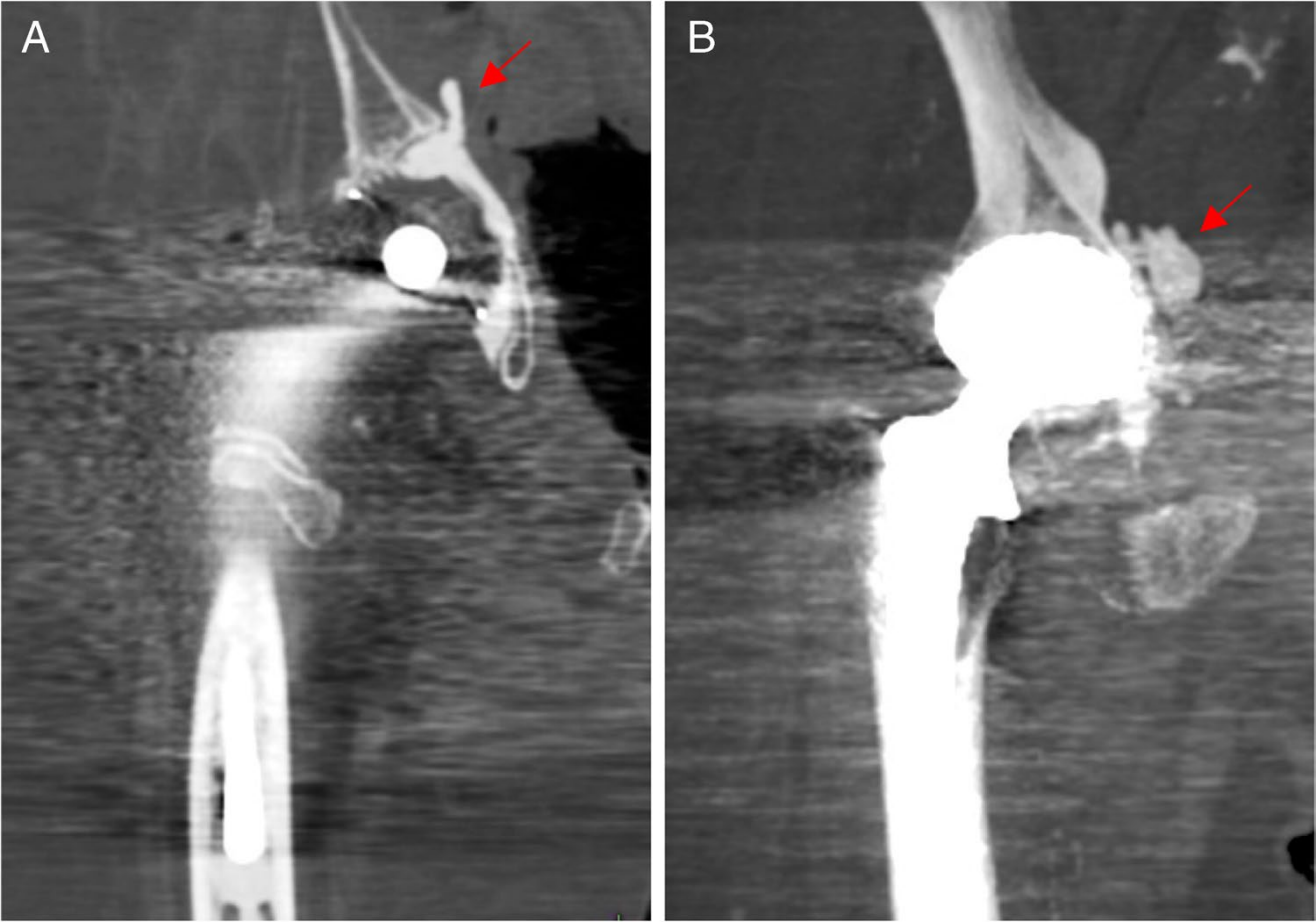
Examples of pelvic CT images with local periprosthetic cement leakage. **A** Needle-shaped cement leakage into the small basin. **B** Oval cement leakage into the small basin. The red arrows point to the cement leakage

Our cohort consisted of 67 patients with a total of 89 joints with cemented total joint arthroplasties (cTJA). Detailed information on demographic characteristics and types of procedures is provided in Table 1. Through advanced analysis on the CT scans, we detected PCE in 46.3% (*n* = 31/67) of the cases. The distribution of PCE grades was as follows: no PCE or grade 0 (*n* = 36/67; 53.7%), grade 1 (*n* = 21/67; 31.3%), grade 2 (*n* = 7/67; 10.5%), grade 3 (*n* = 3/67; 4.5%; Fig. 2A). Since we have already performed a similar analysis in a cohort of patients with cement augmented spine procedures, a comparison with these results was performed. There are only marginal differences regarding the overall frequency of PCE, and the individual PCE grades between the cTJA and the spine cohort (Fig. 2A). Statistical analysis confirmed a similar mean PCE grade between cTJA and spine procedures (*p* = 0.39, Fig. 2B). We also compared subcohorts of THA, TKA, or both and found no differences regarding the distribution of PCE grades or the mean PCE grade (Fig. 2C, D). Of note, we observed local cement leakage in 4 cases of THA (Fig. 3A, B). Of these cases, two cases presented without evidence of PCE (i.e., grade 0), while one case presented as grade 1 and another case with PCE grade 3.

**Table 1:** Demographic overview of the different cohorts including type of procedure and survival time

	cTJA (*n* = 67)	Spine (*n* = 29)	clTJA (*n* = 35)	Control (*n* = 25)
Female/male	47/20	19/10	15/20	15/10
Number of patients (THA/TKA/both)	47/13/7	/	29/3/3	/
Number of prostheses (THA/TKA/both)	62/15/12	/	35/4/7	/
Age at death (± SD)	84.0 yr (± 8.12 yr)	78.9 yr (± 10.80 yr)	74.1 yr (± 12.27 yr)	77.8 yr (± 12.45 yr)
Mean survival	21.0 mo	/	32.7 mo	/

*cTJA*, cemented total joint arthroplasty; *clTJA*, cemented total joint arthroplasty; *yr*, years; *mo*, months

**Fig. 4:**
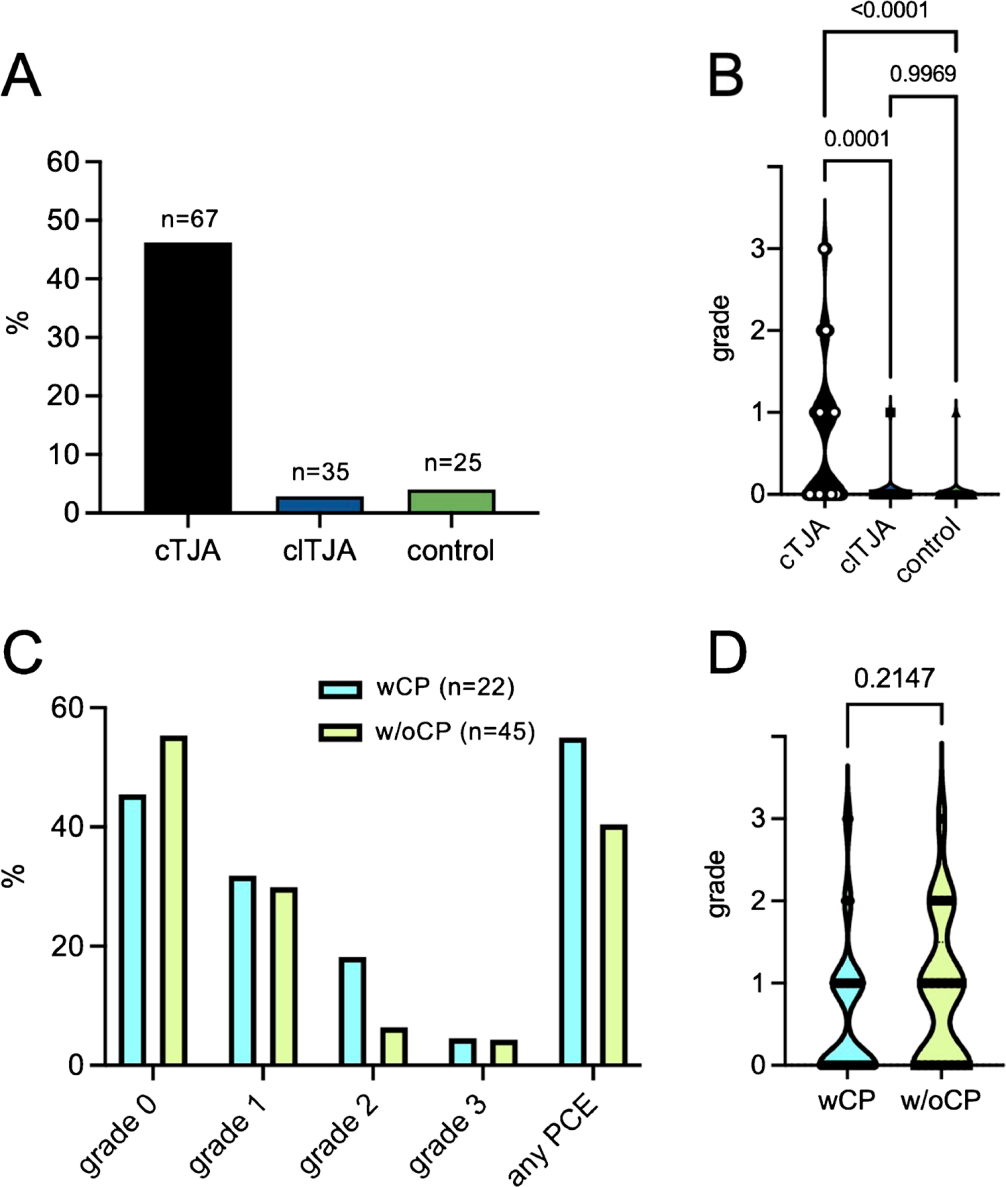
Comparison of PCE occurrence with cementless prostheses and controls and according to the use of cement plugs. **A** Percentage detection of PCE in cTJA, clTJA, and controls. **B** Comparison of mean PCE grade between PCE in cTJA, clTJA. Ordinary one-way ANOVA with Tukey’s multiple comparison test was performed to compare the three groups. **C** Percentage distribution of PCE grades broken down by prostheses with the use of cement plugs (wCP) and without cement plugs (w/oCP). **D** Comparison of mean PCE grade between with and without cement plugs. A *t*-test was used for statistical comparison

To validate our findings, we subsequently compared the PCE occurrence with two different control groups: one group with clTJA (*n* = 35) and one group without evidence of previous TJA (control; *n* = 25). In both control groups, CT findings could almost exclusively be classified as grade 0 (i.e., no PCE). However, we found one case in each group that should have been classified as a grade 1 PCE (Fig. 4A). Statistical analysis confirmed that the mean PCE grade was significantly higher in the cases with cTJA compared to clTJA as well as cases without prostheses (Fig. 4B). Since no bone cement had been used in these control groups, we assume that both cases present small calcified intrapulmonary lymph nodes that cannot be further distinguished from PCE on imaging. Apart from that, age-typical degenerative or calcifying changes (especially of the bronchial trunk) were observed in the remaining cases. We also addressed the question of whether the use of intramedullary plugs/cement plugs (CP) results in a different PCE frequency. In 22 patients, we identified 24 prostheses with CP. They were inserted exclusively into the femur; in 18 patients during THA and in four patients during TKA. There were no differences in the distribution of PCE grades or the mean PCE grade when comparing the cases with (wCP) and without CP (w/oCP) (Fig. 4C, D).

**Fig. 5:**
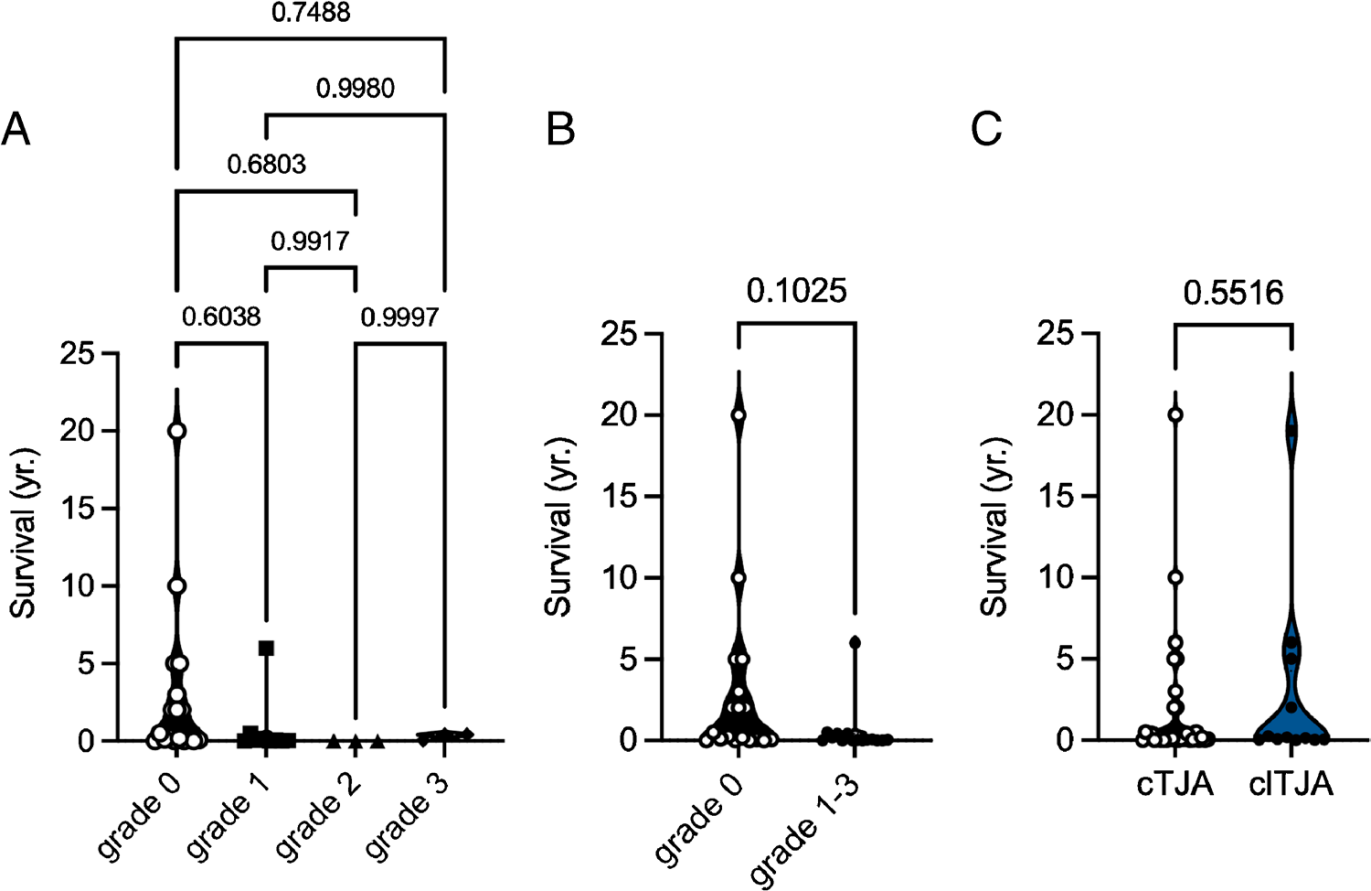
Analysis of the influence of PCE on survival time. **A** Comparison of survival time between PCE grades. Ordinary one-way ANOVA with Tukey’s multiple comparison test was performed to compare the groups. **B** Comparison of survival time between PCE grade 0 and grades 1–3 combined. A *t*-test was used for statistical comparison. **C** Comparison of survival time between cemented TJA (cTJA) and cementless TJA (clTJA). A *t*-test was used for statistical comparison

Finally, using clinical and autopsy data, we were able to determine the time interval between arthroplasty and the time of death in 33 patients. We defined this period, which ranged from a few hours to several years, as the survival time. The mean survival time for the cemented arthroplasty cohort was 21 months. There was no significant difference in survival time for the individual PCE grades (Fig. 5A). Even when comparing the presence of PCE (grades 1–3) with the absence of PCE (grade 0), no significant difference (*p* = 0.1) could be observed (Fig. 5B). In the clTJA group, we were able to determine the survival time of 12 patients, which averaged 33 months. We were unable to determine any difference in survival between cTJA and clTJA (Fig. 5C, Table 1). A conclusive cause of death after an autopsy was determined in 63 patients. None of the autopsy reports listed PCE as a cause of death or a contributing factor in the patients’ death (Supplementary Table 1).

## Discussion

To the best of our knowledge, this is the first study to investigate the frequency of pulmonary cement embolism (PCE) after cemented total joint arthroplasty in a postmortem setting. We demonstrated PCE in 46.3% of the cases in this analysis of 67 CT scans. In a previous work by our group, we were able to demonstrate PCE in 52% and local cement leakage in 69% of the cases after surgical procedures in the spine that involved bone cement [4]. Interestingly, the frequency of each grade of PCE after vertebral cement augmentation is almost identical to the findings presented in this cohort, underlining the relevance of PCE after arthroplasty. The only differences were seen in the occurrence of grade 3 PCE and local cement leakage. We assume that the less severe PCE (grade 3) in arthroplasties can be explained by the anatomical differences between the spine and the hip/knee (e.g., the location to the paravertebral venous plexus, with more voluminous veins, presumably ensures higher-grade PCE). Furthermore, it does not seem to make a difference whether THA or TKA is performed regarding the incidence of PCE. Overall, emboli of lower degree were observed most frequently.

To validate and confirm our results, we compared them with two control groups (clTJA and control). We identified one case in each control group that would have been classified as a grade 1 PCE, although these were most likely to be small, calcified lymph nodes. Bone cement accumulation or migration into regional lymph nodes has been observed in animal studies [7], so smaller lymph nodes with cement accumulation may also show up on CT. Based on these two false-positive cases, we calculated a false-positive rate of 3.4%. Even when correcting the results on PCE in arthroplasty cases, they can still be considered high (i.e., corrected PCE rate of 44.7%). The high incidence of emboli is also confirmed by Hagio et al., who demonstrated embolism by transesophageal echocardiography (TEE) in 61.5% during cemented THA and in only 5.9% of cementless THA [8].

There is already a considerable amount of literature on minimizing PCE risk. Common examples include the placement of a burr hole distal to the prosthesis, flushing fat, and bone marrow from the bone cavity using a jet lavage or inserting a cement plug (CP). The burr hole can be used to lower the intramedullary pressure [9], and thereby reduce the development of embolism and the so-called bone cement implantation syndrome (BCIS) [10]. However, this also reduces the cement distribution into the cortex, which contradicts the recommendations for cementing a prosthesis (maximizing prosthesis retention) [11]. Jet lavage may decrease the risk for BCIS by flushing fat and marrow from the bone cavity [12]. The CP is inserted following jet lavage to achieve a higher intramedullary pressure during cement placement and thus increased penetration of the cement into the bone and maximizing prosthesis retention [11, 13]. Weingärtner et al. reported the absence of distal burr holes as a risk factor for the development of BCIS. In addition, they suggested that CP should be used more frequently as they keep the cement away from the most perfused parts of the bone while not increasing the risk of BCIS. [11]. In the present study, we showed that there is no increased risk of higher-grade PCE when CP are used. It has been repeatedly pointed out that there are neither nationwide standards nor uniform procedures for THA and TKA. A combination of the above preventive measures is likely to help minimize the PCE rate, providing an optimal risk–benefit ratio and prevention of both fat/bone marrow and bone cement embolism. In addition, other ideas, such as the vacuum technique [14] or flushing the medullary cavity with epinephrine [15], have been suggested.

Even though the clinical relevance of PCE is debated [16], and most PCE have been described as asymptomatic incidental findings [6, 17], there are recurrent reports of symptomatic patients [18–24]. When considering the effect of PCE, a distinction should be made between short- and long-term consequences. Short-term events can lead to “mors in tabula” and are often associated with the BCIS [19, 25]. The term BCIS is based on a combination of different explanatory approaches, which is intended to account for the not fully understood pathophysiological mechanism behind the acute intraoperative onset of symptoms after cementation [12].

We were able to detect PCE in almost half of the patients within our postmortem cohort. Even if only grades 2 and 3 were considered definite and relevant degrees of PCE, about 15% of our cohort would still be affected. Of particular interest should also be the long-term consequences of PCE. The risk of cement leakage and/or cement embolism is considered to be of marginal importance in this regard [26], but there are reports of bone cement-dependent complications occurring three or even ten years after surgery [27, 28]. One problem seems to be the migration of cement from the venous system, which can trigger angina-like symptoms due to the perforation of the myo- and pericardium [6, 29, 30]. Therefore, new-onset cardiorespiratory deficits should clinically be considered to be caused by cement embolism or cement leakage [31]. As no reports have been published to date describing pulmonary complications due to migration of the hardened cement, it can be assumed that late cement migrations have mainly cardiac consequences.

However, a late pulmonary complication due to PCE may be an increase in pulmonary arterial pressure (PAD), which has previously also been measured during surgery [32]. Microemboli of bone cement can displace small arterioles (resistance vessels) of the lung so that less than 50% of the pulmonary arteries can be affected to cause a flow disturbance and thus an increase in PAD [33]. Sustained elevation of PAD leads to slow but progressive deterioration of cardiopulmonary function and thus may represent a permanent burden to the heart and lungs even after years [34]. Krebs et al. were able to demonstrate such a permanent PAD increase in sheep after cement application [35]. Dahl et al. measured a difference in concentration of cement components in venous and arterial blood [36]. A lower MMA concentration was found in the arterial samples, meaning that some fractions presumably remain as cement microembolism in the smaller pulmonary arteries. These microemboli are probably not visible on conventional imaging and are confirmed by our high incidence of grade 1 PCE.

The mean survival for the cemented arthroplasty cohort was 21 months, with no detectable differences between cases with PCE to those without PCE. The same applies when comparing the survival time of the group with cemented arthroplasties with the mean survival time of the control group (33 months). It should be critically questioned whether PCE could possibly lead to a significantly lower survival time if a larger number of cases is studied. Overall, since forensic medicine particularly examines postmortem cases in which death is directly related to surgery, this could also result in shorter survival and a more frequent occurrence of PCE than in a clinical cohort. As a limitation, it is possible that the minor differences in age and sex distribution between the groups (cTJA, clTJA, and control) could have influenced the results of this study and may have masked potential differences (e.g., survival). Furthermore, histological studies should examine PCE to confirm the radiological results. In summary, however, it can be stated that none of the autopsy reports listed PCE as a cause of death or a contributing factor in the patient’s death.

## Conclusion

In conclusion, we detected pulmonary cement embolism on postmortem CT imaging in 46.3% of the cases in our cohort after cemented hip and knee arthroplasty. We found no association between PCE and the cause of death. However, the hemodynamic relevance of chronic PCE remains unclear and should be examined in future studies.

## Supplementary Information

Below is the link to the electronic supplementary material.

Supplementary file1 (DOCX 22 KB)

## Data Availability

The data that support the findings of this study are available from the corresponding author upon reasonable request.
