# Supplementary material for: Pulmonary cement embolism is frequently observed but not a contributing factor for death in patients with cemented total hip and knee arthroplasty: a postmortem study
Source: Int Orthop. 2022 Mar 29;46(6):1225–32. doi: 10.1007/s00264-022-05381-6 (PMC9117385; doi:10.1007/s00264-022-05381-6)
Supplement: Supplementary file 1 — Supplementary file1 (DOCX 22 KB) [file 264_2022_5381_MOESM1_ESM.docx]

**Supplementary Table 1:** Overview of the study cohort including the individual causes of death.

| Case | Sex | Age | Cause of death | Grade PCE |
| --- | --- | --- | --- | --- |
| 1 | w | 77 | * | 1 |
| 2 | w | 82 | Bleeding to death | 1 |
| 3 | w | 89 | Heart failure | 0 |
| 4 | m | 85 | Drowning | 0 |
| 5 | m | 86 | Polytrauma | 1 |
| 6 | m | 81 | Cardiovascular failure | 0 |
| 7 | w | 86 | Pulmonary embolism | 0 |
| 8 | w | 88 | Heart insufficiency | 2 |
| 9 | m | 81 | Carbon monoxide poisoning | 2 |
| 10 | m | 94 | Carbon monoxide poisoning | 1 |
| 11 | w | 87 | Myocardial infarction | 0 |
| 12 | w | 57 | Heart failure | 0 |
| 13 | w | 73 | Multi organ failure | 0 |
| 14 | w | 93 | Heart failure | 2 |
| 15 | m | 85 | Cardiovascular failure | 1 |
| 16 | w | 83 | Multi organ failure | 0 |
| 17 | w | 84 | Myocardial infarction | 0 |
| 18 | w | 77 | Polytrauma | 0 |
| 19 | m | 78 | Heart failure | 0 |
| 20 | m | 72 | Heart failure | 0 |
| 21 | m | 95 | Hemorrhagic stroke | 0 |
| 22 | m | 88 | * | 1 |
| 23 | w | 64 | Ruptured aortic aneurism | 0 |
| 24 | w | 86 | Carbon monoxide poisoning | 2 |
| 25 | w | 81 | Subdural hemorrhage | 1 |
| 26 | w | 91 | Heart failure | 1 |
| 27 | m | 84 | Myocardial infarction | 0 |
| 28 | w | 89 | Heart failure | 3 |
| 29 | m | 89 | Pneumonia | 1 |
| 30 | w | 83 | Natural death | 1 |
| 31 | m | 88 | Myocardial infarction | 2 |
| 32 | m | 88 | Myocardial infarction | 1 |
| 33 | w | 86 | Internal bleeding | 0 |
| 34 | w | 89 | Myocardial infarction | 3 |
| 35 | w | 90 | Pneumonia | 0 |
| 36 | w | 91 | * | 0 |
| 37 | w | 92 | Myocardial infarction | 1 |
| 38 | w | 87 | Heart failure and pulmonary embolism | 0 |
| 39 | w | 87 | Heart failure | 0 |
| 40 | m | 82 | Heart failure | 0 |
| 41 | w | 76 | Cardiogenic shock | 0 |
| 42 | w | 85 | Heart failure and pulmonary embolism | 1 |
| 43 | w | 71 | Bronchopneumonia | 0 |
| 44 | w | 89 | Multi organ failure | 0 |
| 45 | w | 94 | Myocardial infarction | 0 |
| 46 | w | 89 | Aspiration | 1 |
| 47 | w | 84 | Sepsis | 1 |
| 48 | w | 74 | Myocardial infarction | 0 |
| 49 | m | 100 | Heart failure and pulmonary embolism | 0 |
| 50 | w | 90 | Sepsis | 1 |
| 51 | w | 87 | Heart insufficiency | 0 |
| 52 | w | 84 | Heart failure | 1 |
| 53 | w | 74 | Pneumonia | 0 |
| 54 | w | 87 | Pneumonia | 0 |
| 55 | m | 79 | Pulmonary embolism | 2 |
| 56 | w | 84 | Myocardial infarction | 0 |
| 57 | w | 98 | Natural death | 0 |
| 58 | w | 82 | Myocardial infarction | 2 |
| 59 | w | 90 | Reclining trauma | 1 |
| 60 | w | 81 | Right heart failure | 0 |
| 61 | w | 84 | Cardiovascular failure | 1 |
| 62 | m | 57 | Polytrauma | 0 |
| 63 | w | 73 | Myocardial infarction | 1 |
| 64 | m | 87 | Myocardial infarction | 3 |
| 65 | w | 90 | Asphyxiation | 0 |
| 66 | w | 88 | Heart insufficiency | 0 |
| 67 | m | 83 | * | 1 |

*Cause of death could not be conclusively clarified. *m* male, *f* female.
